# Safety and efficacy of colistin versus meropenem in the empirical treatment of ventilator-associated pneumonia as part of a macro-project funded by the Seventh Framework Program of the European Commission studying off-patent antibiotics: study protocol for a randomized controlled trial

**DOI:** 10.1186/s13063-015-0614-4

**Published:** 2015-03-20

**Authors:** Clara Rosso-Fernández, José Garnacho-Montero, Massimo Antonelli, George Dimopoulos, José Miguel Cisneros, The MagicBullet study group

**Affiliations:** CTU-HUVR, University Hospital Virgen del Rocío, Avda Manuel Siurot s/n, 787, 41013 Seville, Spain; Unidad Clínica de Cuidados Críticos, Institute of Biomedicine of Seville (IBIS) Hospital Virgen del Rocío, Avda Manuel Siurot s/n, 41013 Seville, Spain; General Intensive Care Unit, Policlinico Universitario A Gemelli, Università Cattolica del Sacro Cuore, Largo A. Gemelli 8, 00168 Rome, Italy; Department of Critical Care, University Hospital ATTIKO, 1 Rimini Str., 12462 Athens, Greece; Unit of Infectious Diseases, Microbiology, and Preventive Medicine, Institute of Biomedicine of Seville (IBIS), University Hospital Virgen del Rocío, Avda Manuel Siurot s/n, 41013 Seville, Spain

**Keywords:** Investigator-driven, Randomized controlled trial, Off-label antibiotics resistance, Multidrug-resistant gram-negative bacilli, Empirical therapy, Ventilator-associated pneumonia

## Abstract

**Background:**

Ventilator-associated pneumonia (VAP) is one of the most common and severe hospital-adquired infections, and multidrugresistant gram-negative bacilli (MDR-GNB) constitute the main etiology in many countries. Inappropriate empiric antimicrobial treatment is associated with increased mortality. In this context, the empirical treatment of choice for VAP is unknown. Colistin, is now the antimicrobial with greatest in vitro activity against MDR-GNB.

**Methods/Design:**

The MagicBullet clinical trial is an investigator-driven clinical study, funded by the Seventh Framework Program of the European Commission. This is designed as a phase IV, randomized, controlled, open label, non-inferiority and international trial to assess the safety and efficacy of colistin versus meropenem in late onset VAP. The study is conducted in a total of 32 centers in three European countries (Spain, Italy and Greece) with specific high incidences of infections caused by MDR-GNB. Patients older than 18 years who develop VAP with both clinical and radiological signs, and are on mechanical ventilation for more than 96 hours, or less than 96 hours but with previous antibiotic treatment plus one week of hospitalization, are candidates for inclusion in the study.

A total sample size of 496 patients will be randomized according to a severity clinical score (at the time of VAP diagnosis in a 1:1 ratio to receive either colistin 4.5 MU as a loading dose, followed by 3 MU every eight hours (experimental arm), or meropenem 2 g every eight hours (control arm), both combined with levofloxacin. Mortality from any cause at 28 days will be considered as the main outcome. Clinical and microbiological cure will be evaluated at 72 hours, eight days, the finalization of antibiotic treatment, and 28 days of follow-up. The efficacy evaluation will be performed in every patient who receives at least one study treatment drug, and with etiologic diagnosis of VAP, intention-to-treat population and per protocol analysis will be performed.

**Discussion:**

Currently, there is no study being undertaken which analyzes empiric treatment of (VAP) with a suspicion of multi-resistance. Colistin, an off-patent antibiotic commercialized for more than 60 years, could widen the antibiotic alternatives for a high-mortality illness aggravated by antibiotic resistance.

**Trial registration:**

This trial is registered with ClinicalTrials.gov (identifier: NCT01292031; registered on 29 June 2012) and EudraCT (identifier: 2010-023310-31; registered on 7 February 2011).

## Background

Infections caused by antimicrobial-resistant bacteria are a serious public health problem, particularly those caused by multidrug-resistant gram-negative bacilli (MDR-GNB). The escalation of resistance is a difficult problem to manage; with resistance to cephalosporins first appearing, more recently followed by carbapenems and finally the appearance and spread of pandrug-resistant bacteria. *Acinetobacter baumannii*, *Pseudomonas aeruginosa* and the *Enterobacteriaceae* are the main MDR-GNB producing serious infections. The treatment of these infections is difficult due to the lack of active antimicrobials [1]. In addition, carbapenem-resistant *Klebsiella pneumoniae* has become a new antibiotic resistance problem in countries such as, Greece, Italy and Spain. This context is likely the best example of the denominated ‘Post-Antibiotic Era’, with relevance even in non-specialized media [2].

MDR *A. baumannii* has turned into one of the main causes of hospital-acquired infection. MDR *A. baumannii* frequently produces hospital-acquired infection and endemic situations in intensive care units (ICUs) all over the world [3,4]. *P. aeruginosa* has a similar trajectory.

Ventilator-associated pneumonia (VAP) is one of the most common and severe hospital-acquired infections. The most common etiologies of VAP are GNB, and among them MDR-GNB are very frequently isolated. VAP is associated with increased attributable mortality, length of stay and use of resources in the ICU [5]. In addition, the risk of inappropriate empiric antimicrobial treatment is higher in areas with a high prevalence of MDR-GNB. The inappropriate empiric treatment of VAP is a risk factor for a poorer prognosis [6-8]. It has been suggested that combined treatment could help improve the results of VAP, since it broadens the antibacterial spectrum [9]. This hypothesis has not been confirmed in a clinical trial that compared meropenem plus ciprofloxacin with meropenem in monotherapy [10]. In many ICUs the proportion of resistance to all the beta-lactams (including carbapenems) and quinolones is so high (over 30 to 50%) [11,12] that empirical treatment with these antimicrobials, even if combined, results in inappropriate treatment in a high percentage of cases.

This scenario of multi-resistance has resulted in colistin being the antimicrobial with greater *in vitro* activity against the GNB causing VAP, including the carbapenems-resistant GNB (CR-GNB) [13]. Unfortunately, the clinical efficacy and security of this ‘old’ antimicrobial have not been thoroughly evaluated. The treatment of pneumonias caused by *A. baumannii* and *P. aeruginosa* with colistin is based on experimental studies [14,15] and clinical series, with variable results [16-19]. According to a non-randomized trial, colistin was less effective and safe than beta-lactams in different infections caused by CR-GNB [19]. However, in this study, groups were not comparable since patients in the colistin group were older, thus coming from healthcare facilities, with ventilator-associated support and/or receiving inappropriate empiric treatment compared to the group treated with beta-lactams. In contrast, other non-randomized studies concluded that colistin has similar efficacy and security compared to beta-lactams [16-18]. It is important to note that the main limitation of these studies is that none of them evaluate colistin as an empiric treatment because it is only administered after the etiologic diagnosis of the infection. Therefore, there is no suitable clinical information on the efficacy and safety of colistin in the empiric treatment of severe infections, including VAP, nor randomized clinical trials that compare colistin with carbapenems or any other beta-lactams.

Another question remaining to be solved is the optimal doses of colistin for the treatment of these infections. Current recommendations are between 3 and 9 MU/day, in two or three daily administrations, based on previous pharmacokinetic and pharmacodynamic (pK/pD) studies conducted in patients with cystic fibrosis [8]. In a recent study in patients with severe MDR-GNB infections without cystic fibrosis, the use of a 9 MU/day dose was an independent factor for positive outcome compared to doses of 3 and 6 MU/day [6]. Recent pK/pD data in critically ill patients recommend the administration of a loading dose, from 9 to 12 MU, followed by doses of 4.5 MU every 12 hours in order to rapidly reach therapeutic concentrations [20].

Infections caused by pan-resistant GNB are the most worrisome problem in the escalation of resistance. There is no effective empirical treatment against these infections. The information available is from *in vitro* studies, experimental studies and some clinical series using colistin alone or in combination with other drugs [21]. In experimental pneumonias caused by pan-resistant *A. baumannii*, colistin combined with rifampicin is superior to monotherapy with colistin [14]. This superiority of the combined treatment has not been demonstrated in clinical trials. Two clinical trials have compared colistin plus rifampicin versus colistin alone, one in patients suffering from VAP caused by *A. baumannii* and the other one including patients suffering from several infections including VAP, with disappointing results [22,23]. In this context of multi-resistance complicating the treatment of VAP, in many centers the definition of the optimal treatment has turned into a public health priority, as has the improvement of the methods for early microbiological diagnosis.

## Methods/Design

The MagicBullet clinical trial is designed as a phase IV, randomized, controlled, open label, non-inferiority and international trial to assess the safety and efficacy of colistin versus meropenem in late onset VAP. This is an investigator-driven clinical study with non-commercial objectives within the general objectives of a global project titled ‘Optimisation of treatment with off-patent antimicrobial agents of ventilator-associated pneumonia (VAP)’ funded by European public competition in the Seventh Framework Program of the European Commission.

The study obtained the authorization of the Spanish Regulatory Authority and the Coordinating Institutional Review Board (IRB) of Biomedical Research in Andalusia (Referral Ethics Committee) (internal approval number 0290–10) which gathered the approval from local ethics committees in all the participating sites in Spain (17 sites): Virgen del Rocío University Hospital IRB, Seville; La Fe University Hospital IRB, Valencia; 12 de Octubre University Hospital IRB, Madrid; Ciudad Real Hospital IRB, Ciudad Real; Jerez Hospital IRB, Cádiz; Dr Peset University Hospital IRB, Valencia; Puerta del Mar University Hospital IRB, Cádiz; Carlos Haya University Hospital IRB, Málaga; A Coruña University Hospital IRB, Coruña; Clinico San Carlos Hospital IRB, Madrid; Santa Lucía University Hospital IRB, Cartagena; Marqués de Valdecillas University Hospital IRB, Santander; Virgen de la Victoria University Hospital IRB, Málaga; Mutua de Terrassa IRB, Barcelona; Orense University IRB, Orense; Juan Ramón Jiménez University Hospital IRB, Huelva; and Reina Sofía University Hospital IRB, Córdoba. The study also obtained the authorization of the Greek Regulatory Authority (72214/10-10-2012 approval number) and National Ethics Committee (NEC Verdict number 73/12) which gathered the approval for all the participating sites in Greece (10 sites): ATTIKO University Hospital, Athens; Sotiria General Hospital, Athens; G Papanikolau General Hospital, Thessaloniki; University Hospital of Larissa, and General Hospital of Larissa, University General Hospital of Alexandroupoli; University General Hospital of Heraclion, Crete; University General Hospital of Ioannina; University General Hospital of Agii Anargiri, Athens; and General Hospital Evangelismos, Athens. For Italy the study has obtained approval from Policlinico A Gemelli IRB, Rome; Federico II University Hospital IRB, Naples; Molinette di Torino University Hospital IRB, Turin with parallel authorisation from the Agenzia Italiana del Farmaco. Three additional Italian Ethics Committees are in the process of evaluation: AO Ospedale Niguarda Ca Granda Milano IRB, Azienda Ospedaliera Sant’Andrea IRB, Rome and AOU Cisanello IRB, Pisa.

The primary objective of the study is to demonstrate that colistin is not inferior to meropenem in the empirical treatment of VAP regarding the primary endpoint (mortality at 28 days of follow-up). As secondary endpoints, clinical healing for intention to treat in clinically evaluable patients and comparison of microbiological efficacy are considered for both treatments arms. The safety of treatment with colistin compared to meropenem in VAP will be followed for all patients.

**Figure 1:**
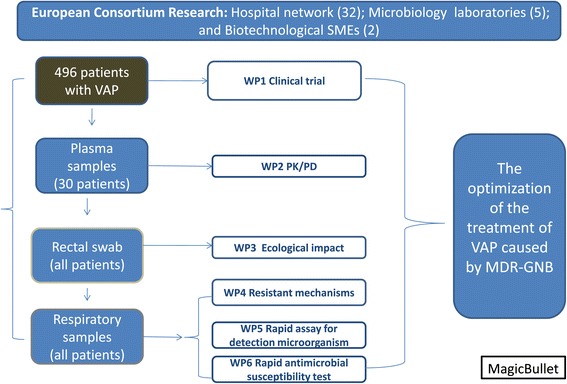
**Workflow of the general project.** SME. Small medium enterprise, VAP; Ventilator associated Pneumonia, WP; work package, pK/pD: pharmacokinetic/pharmacodinamic, MDR-GNB: multidrug resistant gram-negative bacteria.

The clinical trial is one of the work packages of a macro project aimed at seeking solutions to MDR-GNB infections, so that the samples gathered from patients in the clinical trial (respiratory and rectal swaps samples) are re-directed to different collaborative laboratories in Spain (Seville, Barcelona and Coruña), France (Paris) and Germany (Cologne), in order to assess other specific objectives such as the evaluation of the impact of the antimicrobial treatment in the development of antimicrobial resistance and its specific mechanism of antibiotic use on the microbiome. Additionally two clinical centers (one in Spain and another in Greece) also participate in a pK/pD sub-study of colistin. A requirement of European Projects is the participation of small to medium enterprises, two of which are participating in this trial designing and evaluating simple, rapid and reliable procedures to determine antibiotic susceptibility, using a DNA fluorescent staining technique and a novel microencapsulation technology in relevant bacteria isolated from patients with VAP. In addition, a PCR-based technique for the early detection of the microorganism involved in VAP, and its use to measure the ability of antibiotic therapy to clear bacteria from the lung, is being developed as part of the project (Figure 1).

### Selection and enrollment

Patients meeting all inclusion criteria and no exclusion criteria (detailed in Table 1) are candidates for inclusion in the study. In brief, patients with a high suspicion of VAP (by clinical and radiological signs), have been on mechanical ventilation at least for 96 hours or less than 96 hours if they have previously received antibiotic treatment for at least five days and have an in-hospital stay of more than seven days. The setting of the study will be the ICUs of public hospitals in Spain, Italy and Greece.

**Table 1:** **Inclusion and exclusion criteria**

**Inclusion criteria**	**Exclusion criteria**
Age ≥18 years.	Renal insufficiency in substitute treatment.
≥96 hours of mechanical ventilation.	Corporal weight <40 kg or >150 kg.
<96 hours of mechanical ventilation +7 days in-hospital +5 days of antibiotic treatment.	Refractory shock or another disease that, according to the researcher, presents a life expectancy inferior to 48 hours after recruitment.
**Clinical criteria of VAP** (at least one required)	Patients with:
Documented fever	Known or suspected CABP or viral pneumonia
An elevated total peripheral white blood cell (WBC) count (WBC greater than 10,000/mm); or greater than 15% immature neutrophils (bands), regardless of total peripheral WBC count; or leukopenia with total WBC less than 4,500/mm.	Acute exacerbation of chronic bronchitis without evidence of pneumonia
New onset of expectorated or suctioned respiratory secretions characterized by purulent appearance indicative of bacterial pneumonia.	Tracheobronchitis
	Primary lung cancer or another malignancy metastatic to the lungs
	Cystic fibrosis, bronchiectasis, HIV/AIDS, known or suspected *Pneumocystis jiroveci* pneumonia, or known or suspected active tuberculosis
	Immunocompromised patients; hematologic neoplasia, solid organ transplant or congenital or acquired diseases that cause significant immunodeficiency (examples: common variable immunodeficiency), patients with neutropenia <500PMN/mm3.
**Radiological criteria of VAP**: new or progressive pulmonary infiltrate in the thorax radiography which suggests pneumonia and with no other probable cause.	The isolation in respiratory samples from surveillance cultures of GNB colistin or meropenem resistant in the 7 days previous to inclusion.
Modified Clinical Pulmonary Infection Score (CPIS) >4	
Respiratory secretion sample BAL or endotracheal aspirates obtained in the 24 hours previous to the beginning of antimicrobial treatment of the study.	Previous use of meropenem: the current use of meropenem at the time of diagnosis is not permitted (unless this was a unique dose of 1,000 mg as initiation of empiric treatment).
A negative pregnancy test in women of childbearing potential.	
A duly signed informed consent form.	

VAP: ventilator associated pneumonia, WBC: white blood cells, CPIS: Clinical Pulmonary Infection Score, BAL: bronchoalveolar lavage, CABP: Community-Acquired Bacterial Pneumonia, HIV/AIDS: Human immunodeficiency virus/acquired immune deficiency syndrome, PMN: polymorphonuclear neutrophils, GNB: Gram- negative bacilli.

Due to the special conditions of these patients, a specific informed consent form for use in screening period has been approved by all the 32 ethics committees for all sites and countries involved in the trial, allowing the prompt initiation of procedures in the moment the patient meets inclusion criteria, but having obtained consent (candidate or legal representative) while they was being evaluated.

### Randomization

A 1:1 randomization system allows the assignment of treatment arms; colistin or meropenem. Randomization is stratified according to patient severity at the time of diagnosis of the VAP (APACHE Acute Physiology and Chronic Health Evaluation score ≤ or >15) and per clinical site, in order to ensure homogeneous distribution of treatments among sites, and according to the severity of VAP. An automatic system integrated into the electronic case report form (eCRF) permits the inclusion of patients any time of the day or on non-working days. A copy of the randomization list is in the enterprise that manages the CRF in case of technical problems the investigators can proceed with the inclusion of patients just contacting the CRF enterprise directly through a phone call.

### Trial intervention and control

Study drugs are commercial preparations of colistin and meropenem. Colistin is manufactured by G.E.S; Genéricos Españoles, Madrid, Spain. Meropenem is manufactured by Fresenius Kabi España, Barcelona, Spain. The total amount of drugs needed for the study was purchased and specifically labeled for the study following the Good Manufacturing Procedures applicable in the three affected countries. Labeling is provided in the native language for each country. Both drugs were purchased at the beginning of the project in order to ensure the tracking of the investigational medicinal products (IMP) throughout the study as required by Regulatory Authorities. All the authorizations for drug management were obtained in parallel with the clinical trial submission in the three countries and IRBs and ethics committees affected. A specialized warehouse located in Barcelona has been subcontracted in order to perform labeling, distribution and supply for the three countries involved.

Each patient will enter one of the following treatment branches: colistin 4.5 MU intravenous loading dose as infusions of 60 minutes followed by 3 MU administered intravenously every eight hours in 30 minute infusions (arm A); and meropenem 2 g administered intravenously every eight hours in 30 minute infusions (arm B).

Besides the IMPs (colistin and meropenem) all patients will receive levofloxacin which is considered non IMP so that it is provided by the study site pharmacy according the available product approved by its own providers (dose of 500 mg every 12 hours). According to investigator criteria, vancomycin or linezolid are even permitted as empiric treatment if methicillin-resistant *Staphylococcus aureus* is suspected. Any antibiotic prescribed in the 14 days previous to randomization, and any other antibiotic received as concomitant medication (described in List of concomitant medication) once the laboratory results are obtained, will be gathered in the eCRF.

Dose adjustment is detailed in case of renal dysfunction for colistin, meropenem and levofloxacin according to creatinine clearance. For this reason, daily renal function monitoring is performed during the duration of antibiotic treatment. The previous use of meropenem is permitted if there were at least three days of wash-out until randomization in the study.

### List of concomitant medication

Acetaminophen, angiotensin-converting enzyme inhibitors (ACE), acetazolamide, allopurinol, cyclosporine, furosemide, iodinated contrast, immunoglobulins, lithium, mannitol, non-steroidal anti-inflammatory drugs (NSAIDs), omeprazole, penicillamine, thiazide diuretics, torsemide, topiramate, triamterene, tacrolimus and sirolimus.

### Follow-up protocol

A total of 28 days of follow-up are required for every patient included in the study. Treatment assignment is intended for empiric treatment of the VAP, so that once the microbiological results from baseline respiratory samples are obtained, the investigator is free to decide the continuation of the study drug, provided the sensibility is according to the assigned treatment. For this reason the empiric therapy will be adapted to the culture results. As a general rule, physicians are recommended to prescribe a single antibiotic with the narrowest spectrum which has activity against the infecting organism as soon as possible. In the case of isolation of microorganisms only sensitive to colistin and/or meropenem in the culture results, plus good clinical evolution of the symptomatology, the recommendation is to maintain the assigned treatment in the randomization process (colistin or meropenem).

Regardless of changes to treatment, follow-up for patients should be performed at five time-points: baseline, 72 hours, eight days, end of treatment and 28 days. In every visit, patients will be evaluated for clinical status, samples collection and efficacy and safety variables including renal monitoring functioning and adverse events recollection. At the final evaluation, the situation of the patient (dead or alive) and clinical and microbiological results (see Table 2 for descriptions) will be collected.

**Table 2:** **Description of secondary variables**

**Definitions**	**Clinical efficacy**	**Microbiological efficacy**	**Timeframe**
**Cure**	Complete resolution of all signs and symptoms of pneumonia.	Eradication of the bacterium causing ventilator-associated pneumonia during the treatment.	At 28 days of follow-up.
**Failure**	Persistence or progression of signs and symptoms.	Persistence of the bacterium causing ventilator-associated pneumonia during the treatment.	At 28 days of follow-up.
**Relapse or recurrence**	Recurrence of signs, symptoms and/or new radiographic evidence of pneumonia after final treatment.	Recurrence of signs, symptoms or new radiographic evidences of pneumonia present in the last evaluation and isolation of the initial pathogen.	At 28 days of follow-up.
			At any time after treatment finalization to end of follow-up.

### Samples in the study

A respiratory sample taken in the previous 24 hour (bronchoscope with bronchoalveolar lavage or bronchial aspirates) must be obtained before randomization and initiation of any empiric treatment for VAP. The quantity of 10^6^ cfu/mL for the bronchial aspirate and 10^4^ cfu/mL for the bronchoalveolar lavage are considered as cut-off points for positive results. Therefore, samples for the study will be respiratory samples (bronchial aspirates if the patient remains on mechanical ventilation, if not it will be culture of sputum) and rectal swabs, to be recorded at initiation of any empiric treatment for VAP and after 72 hours, eight days, at the end of antibiotic treatment (if different from eight days) and 28 days of follow-up. All study samples will be anonymized, being identified only by the patient study code (site number and patient number), in order to ensure that the association with personal data is not possible. Management of study samples is coordinated from Seville’s Instituto de Biomedicina (IBIS), where all samples are received and distributed to participating centers.

### Outcome measures

The primary efficacy endpoint will be assessed in all patients with evaluable microbiological diagnosis of pneumonia by MITT modified intention to treat analysis (patients with culture confirmed in the relevant sample and who have received at least one dose of the experimental drug or control), by comparing mortality at 28 days between each group.

The secondary variables will include: evaluation of clinical cure in the clinically evaluable population; evaluation of microbiological cure in the microbiologically evaluable population; evaluation of cure, failure or undetermined result at the end of the treatment; evaluation of cure, failure or relapse at the end of follow-up and safety evaluation in all the patients who received at least one antibiotic dose during the 28 days of follow-up.

Safety will be evaluated in patients who receive any of the drugs of the study via an intention-to-treat analysis. Any adverse event occurring from the informed consent form signature to 28 days after the last dose of study medication will be recorded.

### Sample size calculation

The primary efficacy analysis assessed the non-inferiority of the mortality rate of intravenous colistin plus levofloxacin compared to intravenous meropenem plus levofloxacin in the MITT population. Non-inferiority of colistin is concluded if the lower limit of the two-sided 95% confidence interval for the difference in mortality rates (colistin minus meropenem) is ≤10%.

In order to achieve a power of 80% to reject the null hypothesis with a significance level of 5%, assuming as stated in previous studies that the survival rate is 80% with standard treatment [11], and the same with experimental treatment, with a non-inferiority limit of ≤10%, 198 patients are needed in each group. Taking into account that microbiological documentation of VAP is obtained in approximately 80% of the episodes, and therefore about 20% of randomized patients will not have a microbiological diagnosis and will not be useful for evaluation of effectiveness, this requires a 25% increase in sample size. This increases the sample size of intention-to-treat patients included to a total number of 496.

### Statistical analysis

A safety evaluation will be performed in all the patients who have received at least one study treatment drug, independent of reaching the microbiological diagnosis. The efficacy evaluation will be performed in every patient who receive at least one study treatment drug, per MITT. The primary efficacy will first be valued by means of comparison of mortality at 28 days, and secondary variables of efficacy will be valued by means of clinical and microbiological cure.

The clinical evaluation by modified intention to treat (MITT) will be realized in the population that fulfills the inclusion criteria and receives at least one dose of the study drugs. The clinically appraisable population will include those that fulfill the inclusion criteria, who have received the intravenous treatment and with evaluation response data available at the end of the treatment.

### Safety and adverse event reporting

In order to meet all the legal requisitions applicable in clinical trials, a pharmacovigilance system has been implemented for registering, reporting and communicating all adverse events occurring within the patient included in the clinical trial.

Every study team is trained during the site initiation visit on the definitions of adverse events and rules for communication. Any adverse event related or not with the study medication has to be gathered in the eCRF which contains a specific pharmacovigilance module. Only serious adverse events (SAE) are to be completed with more detailed information, such as SAE description (according to MedDra terminology), date of onset and resolution, severity, assessment of causality to study medication, action taken and other concomitant medication and/or procedures. Initial and follow-up communication until resolution is asked of the sites.

The SAEs form is centralized in the CTU-HUVR, whose personnel are responsible for the reception (by fax or email communication), registering and resolution of queries to the sites. A safety medical monitor will assess the SAEs form in order to evaluate if the information is to be communicated to regulatory authorities, ethics committees and investigators, following Good Clinical Practices rules provided for the presence of a serious unexpected adverse event (SUSAR). In cases where a communication through EudraVigilance system is foreseen for any of the three countries this is performed by the CTU-HUVR personnel after the complete information confirmed with the study site and the generation of CIOMS form (Council for International Organizations of Medical Sciences form). Annual safety reports are issued with all the safety information in the study being reported to regulatory authorities and ethics committees (in case of Italy and Greece through the subcontracted contract research organizations (CROs)). The safety medical monitor is responsible for any update in the safety information of the IMPs.

### Study organization

The clinical trial is being performed in 32 reference hospitals in Spain (16 centers), Greece (10 centers) and Italy (6 centers). The coordinating trial site is located in the same public hospital leading the study, CTU-HUVR, and is responsible for the whole coordination of the study and all the sites involved, submitting the administrative authorizations of the study, handling regulatory affairs, contact with ethics committees and response, drug management, labeling and distribution of the IMPs, safety monitoring and pharmacovigilance responsibilities of the sponsor, as well as logistic coordination and providing a contact point for all the 32 clinical teams participating in the study and monitoring activities.

CTU-HUVR acts as a delegation figure of the sponsor (FISEVI, *Fundación Pública Andaluza para la Gestión de la Investigación en Salud de Sevilla*, managing foundation for research in Seville) in relevant activities involved in a multicenter and international trials. In order to ensure the quality of all the activities and resolving specific country aspects related to the approval or local administrative requirements in Italy and Greece, two CROs are subcontracted. Monitoring activities in Spain are performed by clinical research associates (CRAs) pertaining to the Spanish Clinical Trial Network in public hospitals. CTU-HUVR is in close contact with the scientific coordinators of the study, acting accordingly and in a parallel manner so that any decision taken is previously consulted with the study coordination team. Moreover, a project management team at FISEVI is in charge of financial and contractual aspects of the project, as well as general communication and the dissemination plan. A webpage for the study is publically available [24], with specific content requiring login and being password-protected, including the eCRF for the clinical trial, of which managing and updating is the responsibility of the project management team. IBIS-HUVR is in charge of the management of the entire number of samples, including reception, classification and delivery to collaborating laboratories.

### Data and safety monitoring

Remote control of data will be performed through data entry in a weekly fashion by a central CTA working in the CTU-HUVR. Any lack of completion or detected mistake will be promptly communicated to the local monitor (subcontracted CRO for Italy and Greece, and Spanish Clinical Trial network for Spain) in order to be completed or corrected so data are the most accurate and updated as possible. Besides this one site initiation visit per site which provides specific training for the study, monitoring visits (number depending on recruitment rhythm or problems detected) and close-out visit to each site will be performed according to the monitoring plan approved for the study, which details the data to be controlled with source documents, and procedures for the monitoring and reporting of monitoring visits to the CTU-HUVR.

A Data and Safety Monitoring Board (DSMB) is foreseen when 50% of the sample size is recruited. The DSMB is charged with monitoring the accumulating data from the clinical trial to detect and report early evidence of pre-specified or unanticipated benefit or harm to trial participants that may be attributable to one of the treatments under evaluation. The DSMB will conduct an independent objective review of all accumulated data from the clinical trial in such a manner as to maximize benefit to the trial participants and to the research effort. Based on this review, the DSMB shall advise the sponsor on the appropriateness of continuing the clinical trial as designed. In order to duly perform its responsibilities, the DSMB will:

1. review the research protocol and plans for data and safety monitoring;
2. evaluate the progress of the interventional trial, including periodic assessments of data quality and timelines, participant recruitment, accrual and retention, participant risk versus benefit, performance of trial sites and other factors that can affect study outcome;
3. monitoring should also consider factors external to the study when interpreting the data, such as scientific or therapeutic developments that may have an impact on the safety of the participants or the ethics of the study;
4. make recommendations to the sponsor, IRB and investigators concerning continuation or conclusion of the trial; and
5. protect the confidentiality of the trial data and the results of monitoring.

The MagicBullet DSMB monitoring outcome data will be external to the group. Monitoring activities will be conducted by independent experts in all scientific disciplines needed to interpret the data and ensure patient safety. Otherwise, clinical trial experts, biostatisticians, bioethicists and clinicians knowledgeable about the disease and treatment under study should be available if warranted.

### Ethical, deontological and regulatory considerations

The clinical trial will be carried out according to the principles of the Declaration of Helsinki, and according to the legal norm directive 2001/20/EC of the European Parliament and of the council of 4 April 2001 on the approximation of the laws, regulations and administrative provisions of the Member States relating to the implementation of Good Clinical Practice in the conduct of clinical trials on medicinal products for human use.

The trial was not started at any site until having obtained approval of the Ethic committess, conformity of the Directors of the institutions and the authorization of the Spanish Agency of Drugs and Medical Devices (AEMPS, *Agencia Española del Medicamento y Productos Sanitarios*), the Italian Medicines Agency (AIFA, *Agenzia Italiana del Farmaco*) and Greek National Organization for Medicines (EOF, *Ethnikos Organismos Farmakon*). The confidentiality of records that could identify subjects in the MagicBullet trial will be protected in accordance with the European Union Directive 2001/20/EC and as a result of the Good Clinical Practice inspections, in accordance with the applicable national and international requirements and any specific requirement related to data protection in each participating country. All the laws that legislate for the control and protection of personal information will be carefully followed. The identity of patients will not be disclosed in the eCRF; names will be replaced by an alphanumerical code and any material related with the trial, such as samples, will be identified in the same way so that no personal information could be revealed.

As long as the MagicBullet trial will be performed in ICUs, the sort of patients to be included in the trial could be in emergency situations. In cases when prior consent of the subject is not possible, the consent of the subject’s legally acceptable representative, if present, will be requested. A consent form specifically designed to the subject’s legally acceptable representative will be provided with documented approval or favorable opinion by the IRB or EC, to protect the rights, safety and wellbeing of the subject and to ensure compliance with applicable regulatory requirements. The patient or their legal representative must sign a document of informed consent approved by the Ethics Committees as inclusion criteria for the study. Moreover, due to the specific situation of ICU patients, an informed consent form will be offered in the screening period to the representative or relative. This screening period informed consent form has been approved by the Ethics Committees and it is intended to have the possibility of recruitment in case the representative is not easily available (provided the patient accomplishes all the criteria), having the confirmation of the willingness for participation in the trial when the patient is being evaluated.

The Consolidated Standards for Reporting Trials (CONSORT) guidelines will be followed for the reporting of results to any scientific journal or event.

## Discussion

MagicBullet has put in motion one of the first investigator-driven clinical trials of off-patent antibiotics funded by the European Union. In order to initiate this independent clinical trial without the support of the pharmaceutical industry, many tasks had to be developed: selection of the participating hospitals, approval of the trial by the ethics committees of each hospital, authorization of the study and drug management system from the regulatory authorities in the three countries, contracting of two CROs and public procurements for the provision of the eCRF and website, study drugs, transportation of the biological samples and drug handling. For the implementation of all these tasks, great effort was devoted, as international coordination and resources available in public settings are not comparable with the pharmacy industry or private sponsors and CROs. In addition, the project was delayed for several reasons, adding to the difficulties of clinical trials whose recruitment periods are to be extended in most cases, despite the efforts devoted to increase recruitment as described in the literature [25].

This is a multidisciplinary research consortium composed of clinicians with expertise in the diagnosis and treatment of infections due to MDR-GNB; microbiologists expert in the study of the genetics, biochemical and molecular bases of bacterial resistance to antimicrobials; basic researchers expert in human studies and experimental infections by MDR-GNB; researchers expert in industrial research and development projects and experts in clinical trial operations and pharmacovigilance. Through a complete non-commercial network (basically public hospitals and academics) we have designed, have obtained European competitive funding for, and have set up an independent investigator-driven clinical trial among three countries without any pharmaceutical support in order to identify the optimal treatment option for VAP caused by MD-GNB. Moreover, we are contributing to scientific calls such as ‘New Drugs for Bad Bugs’ (ND4BB) [26], on which academic organizations are asking for clinical trials performance to address the problem of antibiotics depletion.

Scientific societies such as the Infectious Disease Society of America (IDSA) and the Food and Drug Administration (FDA) consider the non-inferiority design for clinical trials of patients with VAP to be appropriate, using as the primary indicator the outcome of mortality at 30 days of follow-up, and using a non-inferiority absolute margin of 10%. The MagicBullet trial meets each of these criteria. Scientific societies also recommend that the effectiveness evaluation is performed in the population with microbiological diagnosis and per intention-to-treat analysis. In the MagicBullet protocol, the efficacy evaluation was initially planned to be performed with patients with clinical diagnosis (clinical and radiological) and who were part of the intention-to-treat population. This design has been used for the last antimicrobial approval, doripenem, for indication of VAP [27]. In order to use the same evaluation criteria for efficacy that has been proposed by the FDA and scientific societies, evaluation criteria for effectiveness were modified for efficacy assessment, considering microbiologically evaluable patients per intention to treat (patients with confirmed culture in respiratory sample and who had received at least one dose of experimental or control drug).

Colistin and meropenem are both off-patent antimicrobials. Meropenem is considered as the optimal treatment for GNB causing VAP infections. Colistin, the experimental treatment, has been commercialized for more than 60 years. Up to this project, no randomized clinical trial including such a number of patients with colistin has been carried out.

## Trial status

The sample size recruited to date is 178 patients, accounting for 36% of the sample size (95 in Spain, 36 in Italy and 47 in Greece). The study has been active since May 2012, the date on which the first patient was included. Due to several bureaucratic and local aspects, only recently have almost the total number of sites been active to recruit patients (30 sites now officially opened). The non-commercial nature of this study, entirely performed by public institutions, has faced several impediments which have delayed the activation of clinical sites. Problems such as local administration and approvals, organization of international deliveries or internal organization have caused one year of delay according to the initial planning. During this time we have suffered administrative delays in ethics committees in Italy, delays due to general strikes in Greece and non-reasonable delays due to excessive bureaucracy.

The initial recruitment period was two years, but the different initiation dates in Spain, Italy and Greece and the current activation of the majority of sites, as well as the results obtained by laboratories and the effective recruitment of 36% of the sample size, has permitted the submission of the extension of the project to the European Commission. After the extension, the project will have active recruitment until November 2015, a time we consider it feasible to achieve the expected sample size (495) by, or at least the sample size needed to evaluate efficacy (396).

Activities such as monthly conferences with the active sites, constant communication with investigators and CROs, updated information via monthly newsletters, other specific communications and the external and internal parts of the website of the project have been a key part of the coordination activity. In parallel, onsite and phone meetings have taken place between the CTU-HUVR and the monitors of the clinical trial in order to enhance recruitment at a local level. Recruitment stimulation through personal communication from the study leader, who is responsible for the clinical trial, has been a key factor in the work carried out by the coordination of the clinical trial since its beginning, and will continue in the future in order to achieve the sample size needed for the study.

## Abbreviations

eCRF: electronic Case Report Form
CTU-HUVR: Clinical Trials Unit Hospital Universitario Virgen del Rocío
CRO: Clinical Research Organization
DSMB: Data and Safety Monitoring Board
EC: Ethics Committee
IBIS: Seville’s Instituto de Biomedicina
ICU: Intensive Care Unit
IMP: Investigational Medicinal Products
IRB: Institutional Review Board
MDR-GNB: Multi-drug-resistant gram-negative bacilli
PCR: Polymerase chain reaction
SAE: Serious Adverse Event
SUSAR: Serious Unexpected Adverse Event
VAP: Ventilator-Associated Pneumonia
